# The Use of Photovoice Methodology to Assess Health Needs and Identify Opportunities Among Migrant Transgender Women in the U.S.-Mexico Border

**DOI:** 10.3389/fpubh.2022.865944

**Published:** 2022-05-19

**Authors:** Silvia M. Chavez-Baray, Omar Martinez, Perla Chaparro, Eva M. Moya

**Affiliations:** ^1^Department of Social Work, University of Texas at El Paso, El Paso, TX, United States; ^2^Chicano Studies, College of Liberal Arts, University of Texas at El Paso, El Paso, TX, United States; ^3^Border Biomedical Research Center, College of Science, University of Texas at El Paso, El Paso, TX, United States; ^4^School of Social Work, College of Public Health, Temple University Philadelphia, Philadelphia, PA, United States

**Keywords:** migrants, transgender, Photovoice, health, U.S.-Mexico border, mental health

## Abstract

Psychosocial, social and structural conditions have rarely been studied among transgender women in the U.S.-Mexico Border. This study used Photovoice methodology to empower migrant transgender women of color (TWC) to reflect on realities from their own perspectives and experiences and promote critical dialogue, knowledge, and community action. Sixteen participants documented their daily experiences through photography, engaged in photo-discussions to assess needs and identify opportunities, and developed a community-informed Call to Action. Four major themes emerged from the participants' photographs, discussions, and engagement: (1) mental health, (2) migration experiences and challenges, (3) stigma, discrimination, and resiliency, and (4) impact of the COVID-19 pandemic. Through active community engagement, a Call to Action was developed. A binational advisory committee of decision makers and scholars reviewed a set of recommendations to better respond to the needs of TWC in the U.S.-Mexico Border. Photovoice served as an empowerment tool for TWC to assess the myriad of syndemic conditions, including mental health, stigma, discrimination and COVID-19, affecting them daily and identify initiatives for change.

## Introduction

A growing body of literature has started to document the growing health disparities and inequities impacting transgender women of color (TWC) (1–5). TWC encounter structural barriers related to having appropriate access to health care, limited opportunities to a formal employment, structural violence, victimization, discrimination, migration, and housing instability (6–10). In particular, migration is a public health concern affecting TWC in the U.S.-Mexico border region. The migration journey can exacerbate vulnerabilities and health inequalities in countries of destination, provoked by hard living conditions and economic deprivation of individuals and family experiences (11). TWC who flee to the United States (U.S.) through the U.S.-Mexico border may be subjected to sexual, physical, verbal assaults and robbery. TWC are often the target of abuses by smugglers, gangs, and government officials along the migration journey and face a dire situation of health threats and negligence at immigration detention centers (12, 13). A study, conducted prior to the COVID-19 pandemic by the United Nations High Commissioner for Refugees (UNHCR), reported that 88% of LGBTQI+ asylum seekers from the Northern Triangle countries (Honduras, Guatemala and Salvador) were victims of sexual and gender-based violence in their countries of origin and suffered similar attacks in Mexico fleeing poverty, violence, and persecution (14). Further, transgender asylum-seeking individuals are often exposed to traumatic events in their country of origin, during the migratory journey, and post-migration. Among the migrants forced to flee, transgender individuals are among the most vulnerable in the world. In particular, Latin America's persecutory transphobic laws frequently target transgender individuals and policies often resulting in torture, human rights violations, human trafficking, and death (15). Thus, there is an urgent need to further understand the migration experience among TWC and its implications for public health and service provision.

Across law, education, and government venues, trans people are rendered invisible, illegible, or disallowed through cisnormative systems that disregard identities that do not adhere to the gender binary. Because the cumulative intersectionality of oppression, multiply marginalized trans people represents those facing individual and structural mistreatment, discrimination, violence, and economic hardship between trans people of color, with irregular status, and disabilities, among others. Lack of protection and inclusion in public policy results in a need for services, advocacy and specialized care. The legislative practices demonstrate the ways in which the abandonment and unjust treatment of the transgender community extends beyond the irrefutable discourse (16).

Researchers have also documented the disproportionate impact of psychosocial conditions among TWC including critical gaps in HIV prevention and care, substance use, depression and anxiety, suicidal ideation, and interpersonal physical and sexual abuse (17–19). For example, it is estimated that Black (44.2%) and Latina (25%) transgender women have a higher prevalence of HIV compared to White transgender women (6.5%) (20). Thus, the need to increase appropriate trans specific HIV prevention and care services, as well as increasing availability of gender affirming treatments. Further, disparities in suicide risk and intimate partner violence are higher in transgender women (17, 21). Despite this research, little is known about the unique needs and barriers affecting transgender populations in the U.S.-Mexico border region.

The COVID-19 pandemic has also exacervated preexisting inequities and health disparities and inequities among TWC (22–24). Under president's Trump administration, the U.S. government invoke Title 42 of the Public Health Service Act, closing the U.S.-Mexico border to asylum seekers, non-essential travelers and implementing express deportations. These provisions targeted migrants and continued with deterrent practices such the Migrant Protection Protocols (MPP), known as Remain in Mexico (25). As a result, migrants had to find temporary housing, shelters, and refugee camps. Immigration procedures were put on hold and vulnerability to organized crime became more prevalent (26). It is also essential to note that stressors related to uncertainty can also have a negative impact on immigrants' mental health (27).

Structural frameworks have started to emerge to better advance the rights and protections of transgender individuals, including TWC who are often further marginalized and structurally and systematically excluded. Trans justice is guided by the pressing needs of marginalized transgender people and go beyond legal recognition through explicit human rights grounds to helping survive by increasing access to low-cost housing, social benefits, fighting against racial profiling, supporting transgender parents and youth, and through other projects to increase life chances (28). Respect for the rights of dignity and equality is grounded on the right to be free throughout the life cycle and equal in dignity, where no one is superior to another, with same rights and opportunities, without discriminating against anyone because of creed, faith, skin color or sex, among other aspects. One of the pillars driving inequities among transgender populations is the lack of universal rights including adequate medical and psychologically services, legal protections and respect of human dignity (29).

This study outlines the results of a project titled Use of Photovoice to Make Visible the Mental Health of Migrant Transgender Women in Juarez, Mexico and El Paso, Texas. The Photovoice methodology served as a means to empower transgender women to record and reflect on realities from their own perspectives, thus promoting critical dialogue and knowledge about individuals and community issues. The ultimate goal is to facilitate social change through group discussions capable of influencing policy and decision makers. Instead of being passive in the research process, participants become actively engaged in recording their own experiences as co-researchers who collect data, frame discussions, and conduct the analysis.

## Methods and Materials

### Photovoice as a Participatory Action Research Method

We utilized the Photovoice method to collect qualitative data presented in this study. The method is based on five concepts: (1) images and stories provide both insight and a learning opportunity, which in turn influence individuals' health and well-being; (2) images and stories affect policy by influencing the way we look at the world and the way we see ourselves and therefore have the potential to influence decision makers and the broader society as well; (3) individual community members actively participate in creating and defining the images and stories that shape public health policy; (4) the involvement of policy and decision makers, and particularly community stakeholders who can affect change, from the beginning of a study is central to success; and (5) both individual and community advocacy efforts aim at mobilizing and empowering participants are required to effect social change (30–32).

Photovoice is a Participatory Action Research (PAR) method that employs photography, narrative, and group dialogue to deepen on a community issue or concern, integrating community and individual approaches to the use of photographs and narratives to produce knowledge and social action (30, 33).

### Institutional Review Board

The University of Texas at El Paso Institutional Review Board approved the study as a binational exploratory cross-sectional mix-method with a qualitative orientation to investigate transgender migrants' mental health and the impact of COVID-19 in Juarez and El Paso.

### Sampling, Recruitment and Engagement

Study participants consisted of 16 adults (*n* = 16). Data was collected between May 8 and July 30, 2021. We used a multi-strategy recruitment approach. The investigators used a convenience sample and recruited participants from two local shelters in Ciudad Juarez (Casa de Colores and Casa RESPETTTRANS), both serving LGBTQI+ immigrants and asylum applicants. We also recruited participants from Familias Triunfadoras, a community-based organization working with migrants and victims of gender violence, in El Paso, Texas. Snowball sampling was also integrated in the recruitment approach to identify participants based on similar characteristics and pre-established criteria for recruitment (i.e., transgender women with history of migration) (34). Inclusion criteria consisted of adults 18 years and older, history of migration in the past 7 years and self-identification as transgender. The original recruitment consisted of 26 participants, 18 received a project orientation, and 16 enrolled and consented to participation and completed the project. Participants received a stipend of $25 dollars as well as educational materials (e.g., brochures, and health materials) for each session they attended.

The group sessions initiated in Juarez, Mexico using Zoom and in-person modalities in shelters in study location. Once the project participants crossed to the U.S., sessions were hosted face to face and using Zoom at a local shelter and then at the university campus to connect participants who moved outside of study location.

### Participant Characteristics

Even though all participants identified as transgender at the beginning of the project, when completing the registration form, 12 (75%) identified as transgender women, 2 (13%) as binary, 1 (6.5%) as female and 1 (6.5%) as cross-dressed. Participants reported 13 (81%) being single, 2 (13%) Common Law and 1 (6.5%) married. The age range consisted of 18–45 years old, mean age of 27. Their education attainment were 1 (6 %) elementary school, 4 (25%) middle school, 7 (44%) High School, 2 (13%) technical degree, and 2 (13%) University degree. Ten participants (63%) migrated to U.S. from El Salvador, 2 (13%) Honduras, 2 (13%) Mexico, 1 (6 %) Guatemala, and 1 (6.5%) U.S.-Mexico border. Once they were able to cross to El Paso, 7 participants migrated to cities in Texas (44%), 3 to Iowa, (19%), 3 to New York State (19%), 1 to Washington State (6 %), and 1 to California (6 %).

### Participatory Analysis Using Critical Reflection and Dialogue

As part of the methodological approach used, the project's facilitators (primary, third and senior authors), research assistant/observer, and participants were included in the data analysis. The experienced researchers reviewed the group digital/audio recordings and transcriptions to capture the major elements and themes. Participants engaged in a three-pronged process of participatory analysis consisting of selecting photographs that most accurately reflected their realities and concerns, contextualizing through narratives about the meaning, and codifying the issues and themes that emerged from their stories. The process is grounded on critical consciousness (35) as well as concepts from Feminist theory (32, 33).

Each participant selected two to three photographs per session that were the most significant or liked best; they contextualized them through group discussions; shared stories about them and defined the meaning of images. The discussions were in Spanish and the narratives produced by participants were translated to English and back translated to Spanish by a bilingual graduate research assistant and reviewed by the director of the translation service office. Participants framed their stories in terms of questions spelling the acronym SHOWeD: What do you **S**ee here? What is really **H**appening here? How does this relate to **O**ur lives? Why does this problem or strength **E**xist? and What can we **D**o about it? (36). These questions were used to probe for deeper understanding during the individuals' sharing of photos and narratives. Participants carried consent forms and phone cameras with them for a period of 4 weeks and were asked to take three to six photos per week. After orientation, each participant selected two or three photos to share with the group at subsequent meetings. The framing questions led the participants to identify areas of opportunities and resources, critically discussing the determinants of their situation to develop recommendations and strategies to change the situation (37). We used pseudonyms to protect participants identities and confidentiality.

## Results

Each photograph is accompanied by text developed by individual study participants to describe their photo.

### Mental Health

The first round of photos was framed on describing their own mental health status, community and surroundings. They talked about emotional ambivalence because of the migratory journey, what they leave behind, and the emotions and experiences lived in different shelters. Participants described challengnes and mental health experiences and identified loneliness, sadness, happiness and support they received by shelters in Juarez and El Paso as well as the sufferings and loss associated with forced migration. Further, during their narrative reflections participants described experiencing frustration, anxiety, and panic attacks in some cases. In one instance, a participant voiced, “Sometimes it was two or three in the morning, and they would wake me up because someone was collapsing from everything that was happening. I felt a big responsibility and I would crumble; it was horrible. Everybody was crying”.

#### Solitude and Happiness

**Figure 1:**
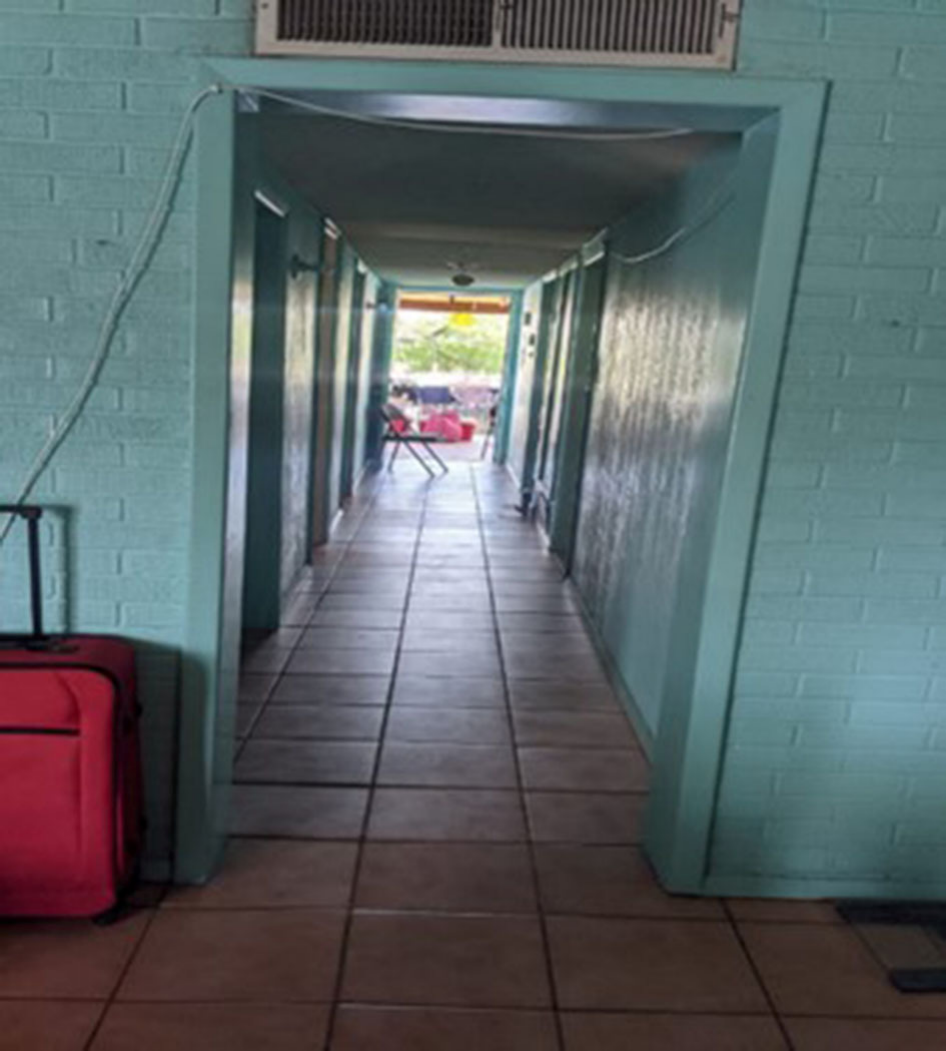


“*This photograph represents the time I spent in Ciudad Juárez in Casa de Colores (shelter). I met and lived with the trans girls. Later the shelter looked more alone, I walked the entire hallway and the upstairs area and there was hardly anyone left. I felt sadness and at the same time happiness, because every time we were all accomplishing what we wanted, to enter the United States. It is something very moving and happy. An experience, a moment that I lived happily, because I met many people”* (Figure 1).

Keyra, from El Salvador

#### Solitude

**Figure 2:**
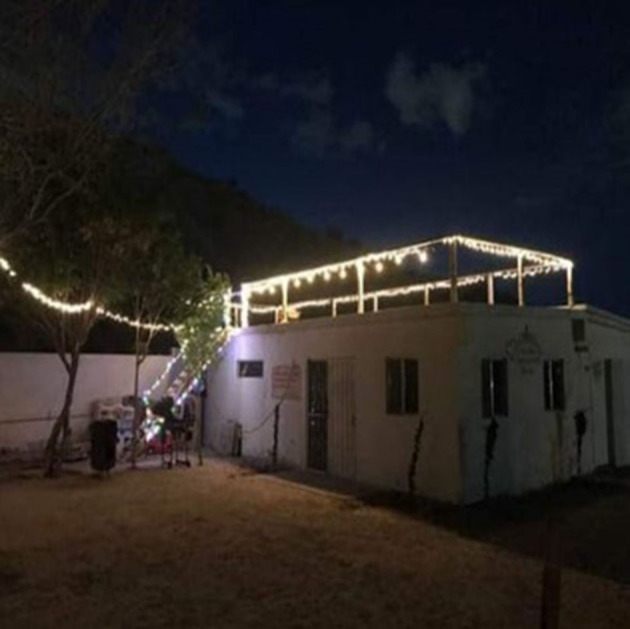


“*I see a pretty place (shelter) that helps a lot of people that truly need it; it is in Anapra (Juárez). The decoration was during Christmas time, that is why it is so lit up and strikingly colorful. It relates to us because we spend many afternoons there with friends, enjoying a refreshment or a cigarette. This place exists to help people who are migrants and who need it. When we arrived in this city, we did not have anywhere to live. In my opinion I'd say it is worth supporting these shelters”* (Figure 2).

Mane, from Honduras

### Migration Experiences and Challenges

The second round of photos and narratives focused on the challenges attributed to migration experiences. Participants reflected on the challenges of leaving family behind in their country of origin and the impact of family separation. The border is seen as a “wall” separating families and friends. Others reflected on the liberation resulting from the migration experience, as one participant articulated, “*you can be whoever you want to be.”* Many recounted the traumatic experiences of discrimination in their home country, for many resulting in death.

#### The Other Side

**Figure 3:**
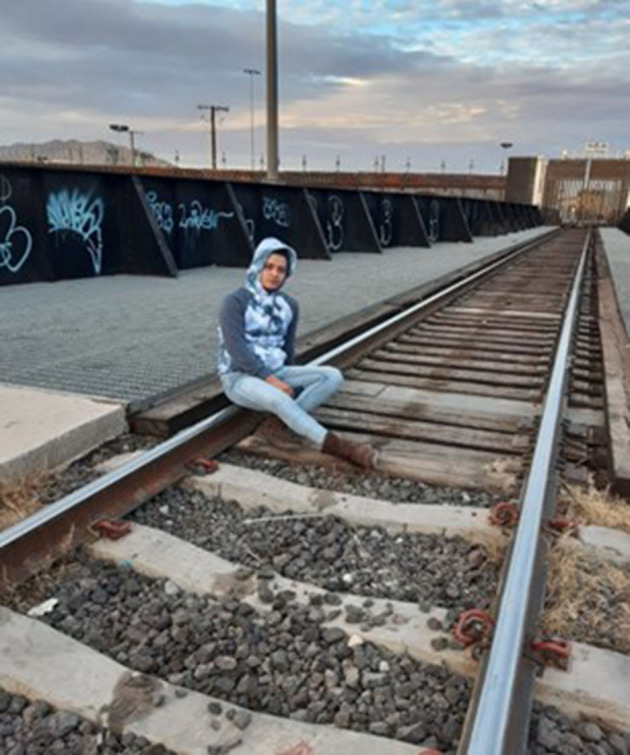


“*I desired so much to get to this side (United States); it was extremely emotional to be able to see the city, being on the other side. At the same time, how many people have left their blood thrown on the floor just to accomplish what I have reached, and wanting to throw down this wall, it is very sad at the same time because many have attempted it, how many people and families have been separated? If it were up to me, I would say, take it down, that wall cannot separate us, it cannot separate the family, it cannot separate friends. A wall cannot separate the family nor our willingness to thrive”* (Figure 3).

Tanya, from El Salvador

#### Not Just a Shoe

**Figure 4:**
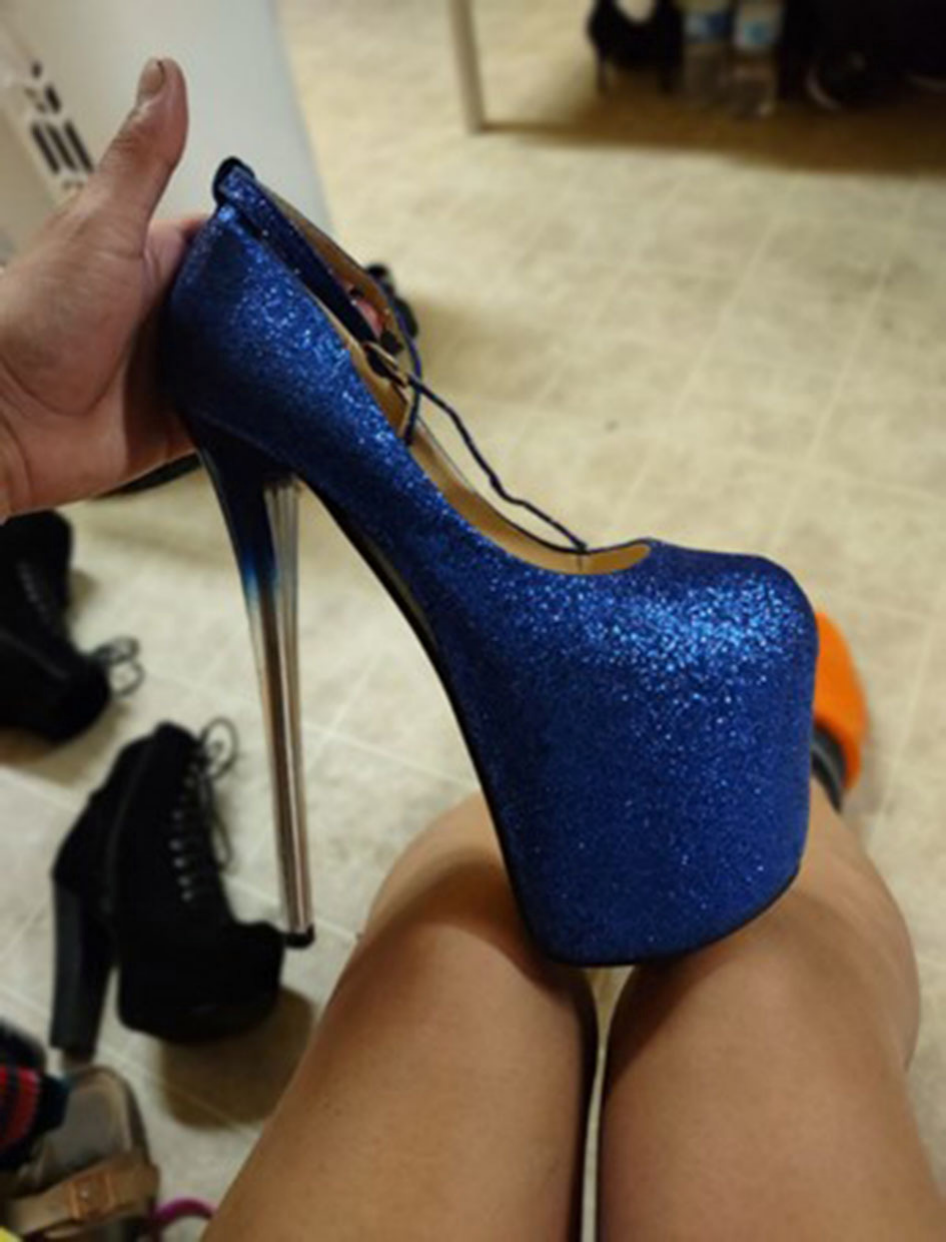


“*This photo was sent to me by a friend. When I saw it, I was reminded of the first time I put on heels and how badly I wanted to be myself when I was living in my home country. Sometimes when you walk through stores in our home country and normally go as a man, you are only able to see the things you like but not take them with you. It is like a very odd disability, knowing what you want and can have, but you cannot truly wear it because a simple shoe is like a symbol of death and discrimination [reference to violence from gangs], or getting beaten and treated poorly. An image of a shoe brings back memories. It reminded me of the first time I was able to put on heels and dress as a woman, and it brought newfound emotions because I remembered good things, when I had fun times, what it made me feel while at the same time it made me remember the bad things that happened. I mean, they use to happen because now I live in a new country (United Stated) which I think supports us a lot and we can be who we want. My friends laugh because when they ask me if I am trans, I tell them I am Barbie because as the motto says, you can be whoever you want to be”* (Figure 4).

Valeria, from Honduras

### Stigma, Discrimination, and Resiliency Among Transgender Women

Participants described how stigma associated with gender identity resulted in discrimination, lack of opportunities to study and find the job they desire; dress as they chose resulting in violence and life threats against them. In the first 2 weeks of their arrival to the United States, they expressed feeling a sense of safety, protection and freedom to dress and be themselves.

#### Raise Your Voice

**Figure 5:**
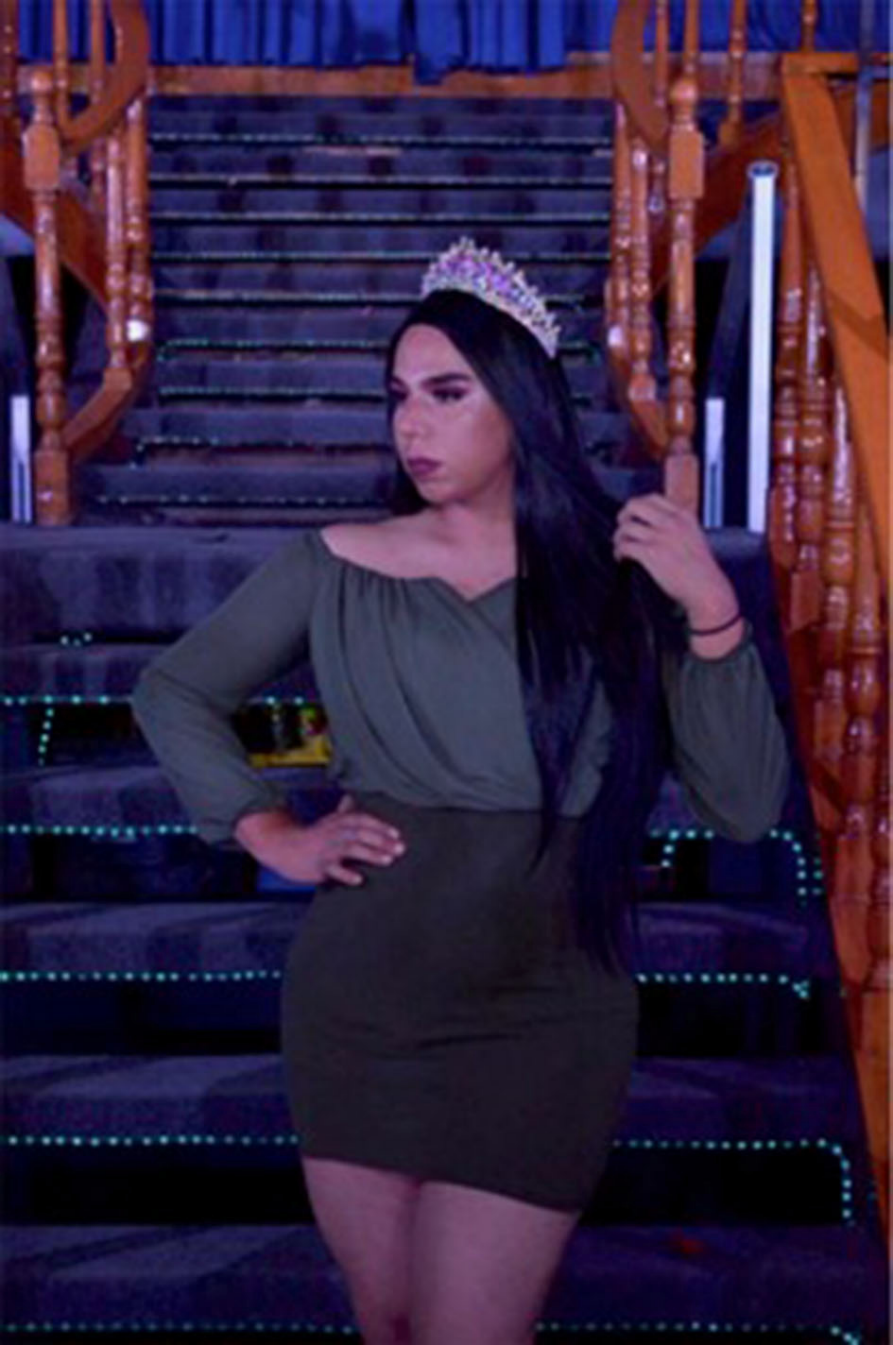


“*My name is Shay Dominguez, and today I am utilizing my image as a trans girl and as a beauty queen representing my county (Mexico) to raise my voice and say, “I fight for my right to be respected and I also offer it to you; I fight for equality.” Being the queen does not only signify you are pretty, it signifies being a female leader, a good representative of the community; it also signifies using your platform to create a change within decision makers. I would like for you to hear what we want and need and be able to give you an explanation of why trans women suffer day by day discrimination, sadly we are not well accepted in society. Trans women are strong, we need support, and if those of you reading this do not give it to us, who will? It is time to speak out and never be silenced again”* (Figure 5).

Shay, from Transborder, Mexico U.S.

#### New Project of Life

**Figure 6:**
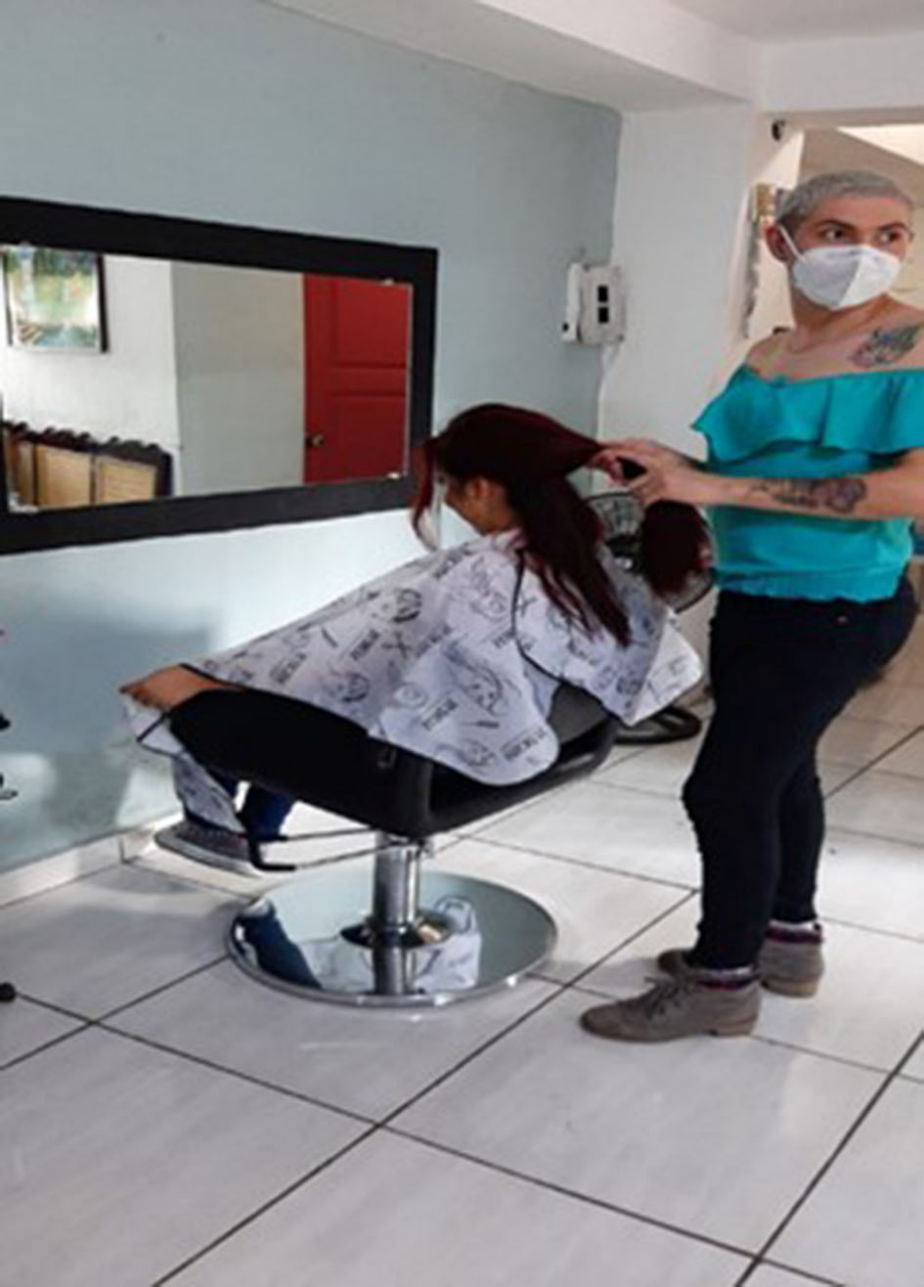


“*One of the trans girls and most of them, due to not having studied, dedicate their work as stylists, it's what most of the girls look for. Some do it because they like it and others because they have not had the opportunity to study due to being trans girls”* (Figure 6).

Mayte, from El Salvador

### Impact of the COVID-19 Pandemic

The fourth round of photos and stories focused on emotions and the effects of the COVID-19 pandemic. All participants mentioned they had COVID-19 during their stay in a shelter in Juarez in early 2020. They described the fear and suffering experienced as result of being in quarantine and not having information and medication. A participant described how a transgender friend died close to her at a U.S. detention center. Another participant shared the loss of the younger sibling and her frustration and sadness for not being able to return to the country of origin to be with her family. Another component impacting their mental health was the impact that COVID-19 had in their plans to migrate, find employment, housing, life without violence, and in some cases move forward with their gender reaffirming health care. As one of the participants pointed out, “We felt trapped, without being able to send money to our families, without being able to move forward, nor backward (due to the pandemic).” In addition, they expressed hope in improving their quality of life for being in the U.S. for the first time.

#### Angel

**Figure 7:**
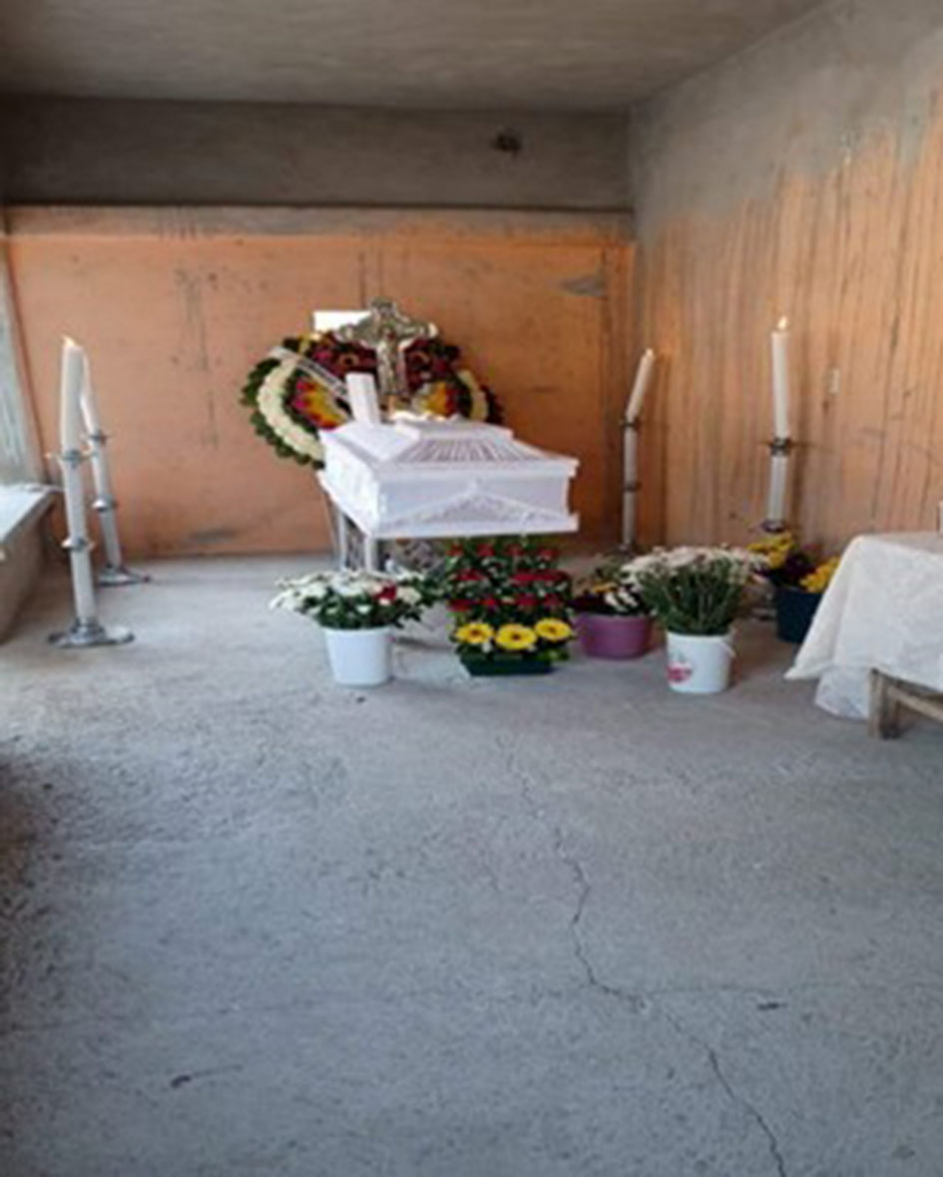


“*Migrating is usually a synonym of wellbeing but deep inside exists great sacrifices that you must face and that hurt more than anything. You leave your house without knowing if you will ever see the most important people in your life ever again. Without knowing if the last hug you gave them would be the last, you ever had; the last kiss, the last look in those teary eyes that said: I love you, take care of yourself. I recommend that during your goodbyes, you try and enjoy that last hug a lot more”* (Figure 7).

Ariel, from Mexico

#### Consequences of the Pandemic. Closing of the Border

**Figure 8:**
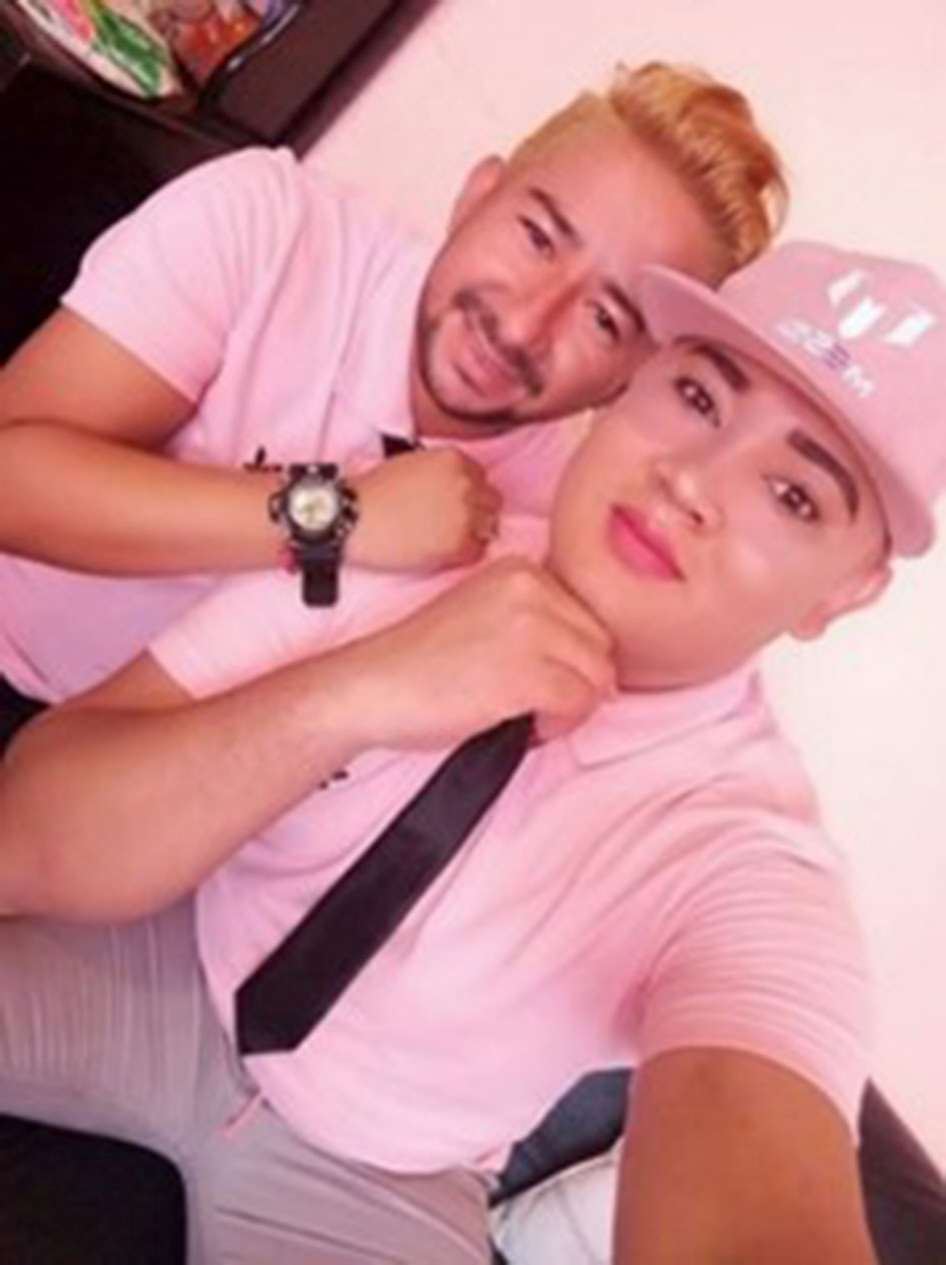


**Figure 9:**
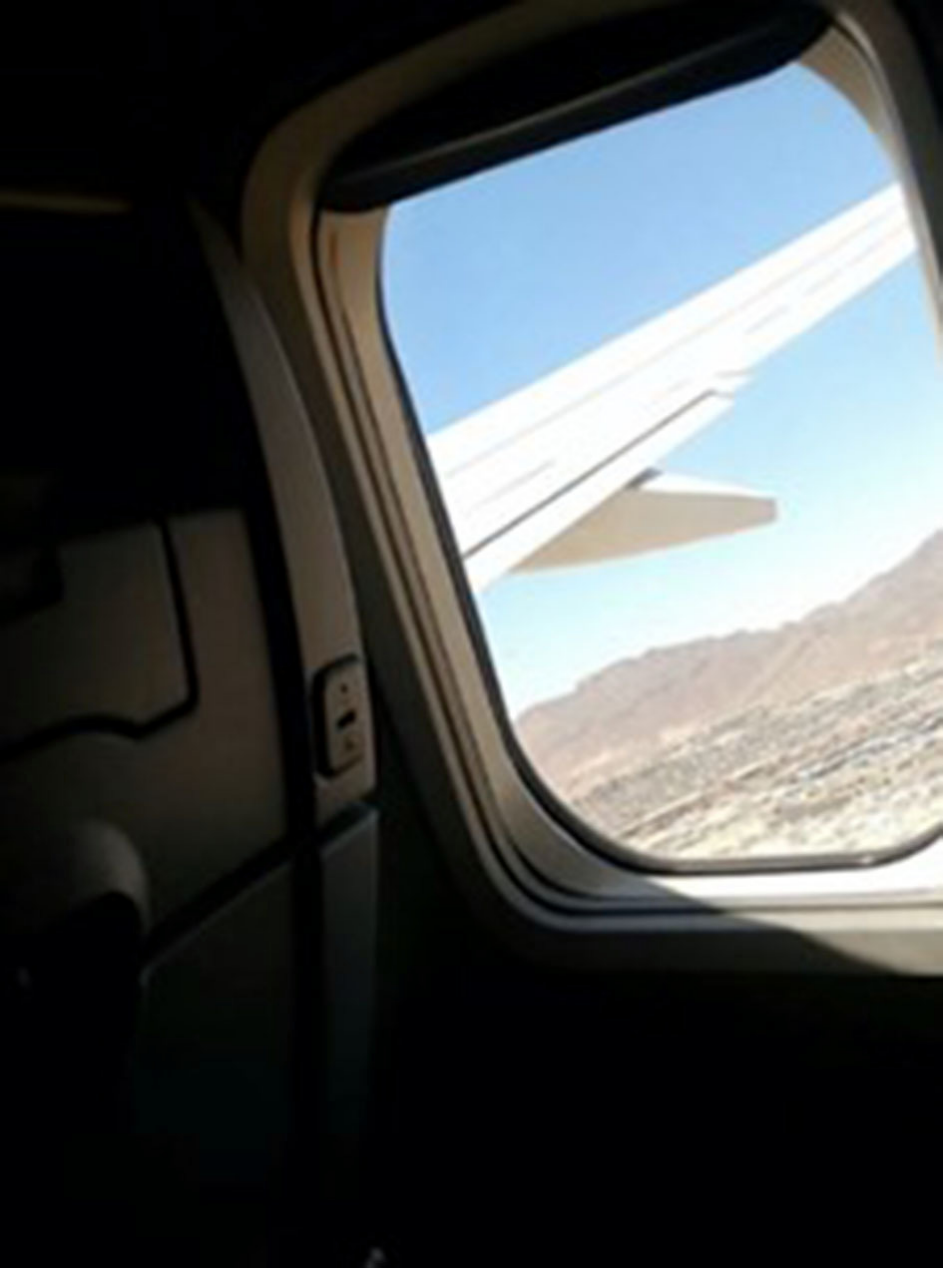


“*This was a very special day since it had been such a long time that I had been in lockdown in my country due to the start of the pandemic, where it affected my plans of traveling; it was disappointing getting the news of the border closure. As soon as I heard the news of the border opening on September 22, I decided to leave my country because I so badly wanted to be back in Mexico. I felt great emotion to return to Mexico, where I felt safe without running the risk of danger, being with people who were not my family, because they are very important in my life, where they demonstrated their friendship, trust, affection, what I value and the happy moments. I feel blessed and grateful with God for always putting people with big hearts in my life. Living and experimenting every moment, does not have a price. I am very grateful to all the people that were part of my dream. I am so excited to leave it all behind, primarily the obstacles that destiny put in my way, I was able to do it (arrive in the United States). One of my dreams came true and there are many more that I will accomplish. It excites me, meeting new people, like the ones I am meeting at my work and a group of people who are amazing. I am excited and blessed”* (Figures 8, 9).

Keyra, from El Salvador

#### I Don't See a Border, I See Hope

**Figure 10:**
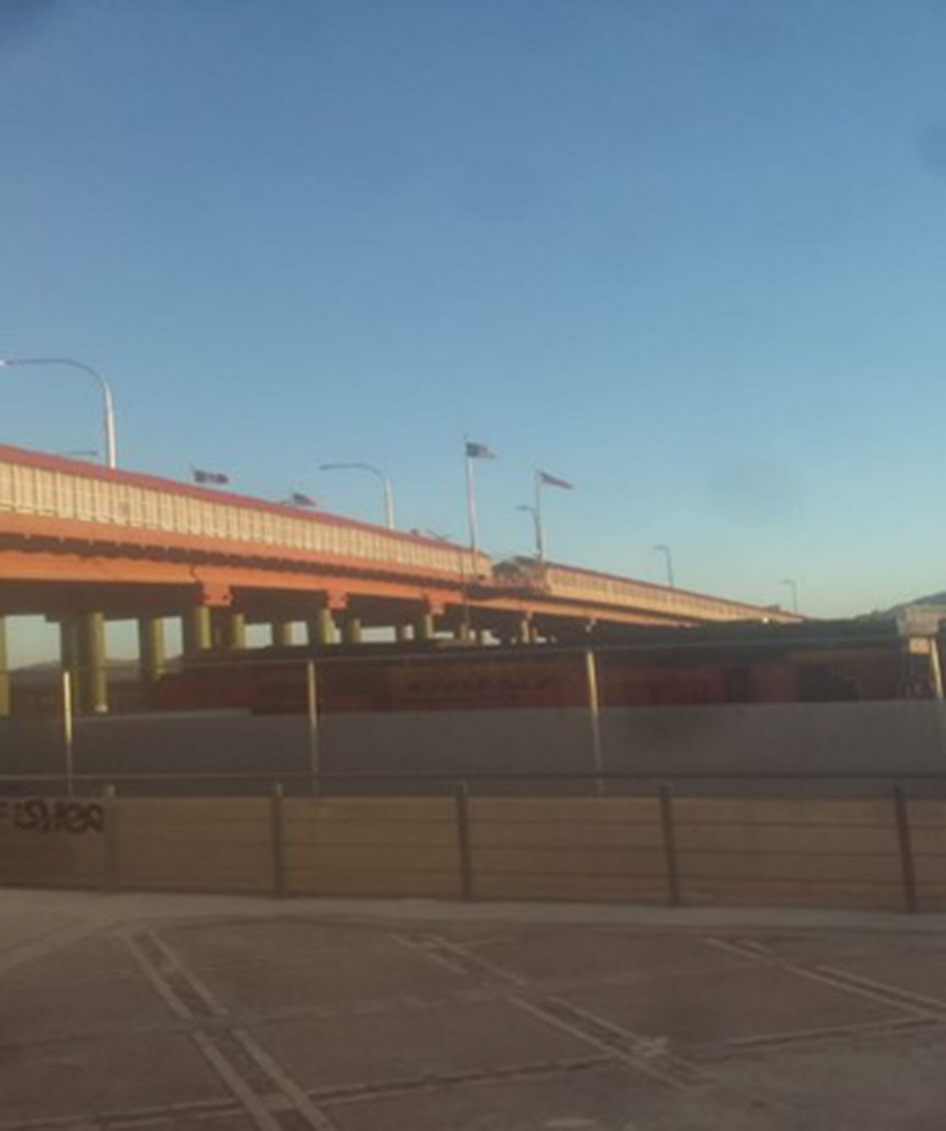


“*A border that divides what has been and what will be. The happiness that you see before you and what you leave behind. How much can someone's life be changed after they have suffered for such a long time? A line of hope and comfort, as you cross through lines you get closer to happiness. You receive the rejection but with the promise of being happy. A country (United States) that promises you happiness, comprehension, and less pain. Another change that gets you closer to the person you want to be, without being judged for our appearance or identity. And when I cross that line, that barrier that border and I look in the mirror, I don't see what was…I see the hope of a better future”* (Figure 10).

Iran, from Guatemala

### A Call to Action

By the fourth week, most participants had left El Paso to meet with their potential sponsor. The last round of photographs and narratives described actions and recommendations. Some of the participants indicated that the American dream was shattered because the freedoms and rights aspired were not found. They commented that they had faced discrimination in the workforce, racism, lack of identification or recognition, inability to access basic health care services and fear of becoming ill and not being able to receive care due to lack of medical insurance and financial resources. Regardless of the challenges and inequalities encountered, they expressed feeling safe in the U.S.

#### The Oppression

**Figure 11:**
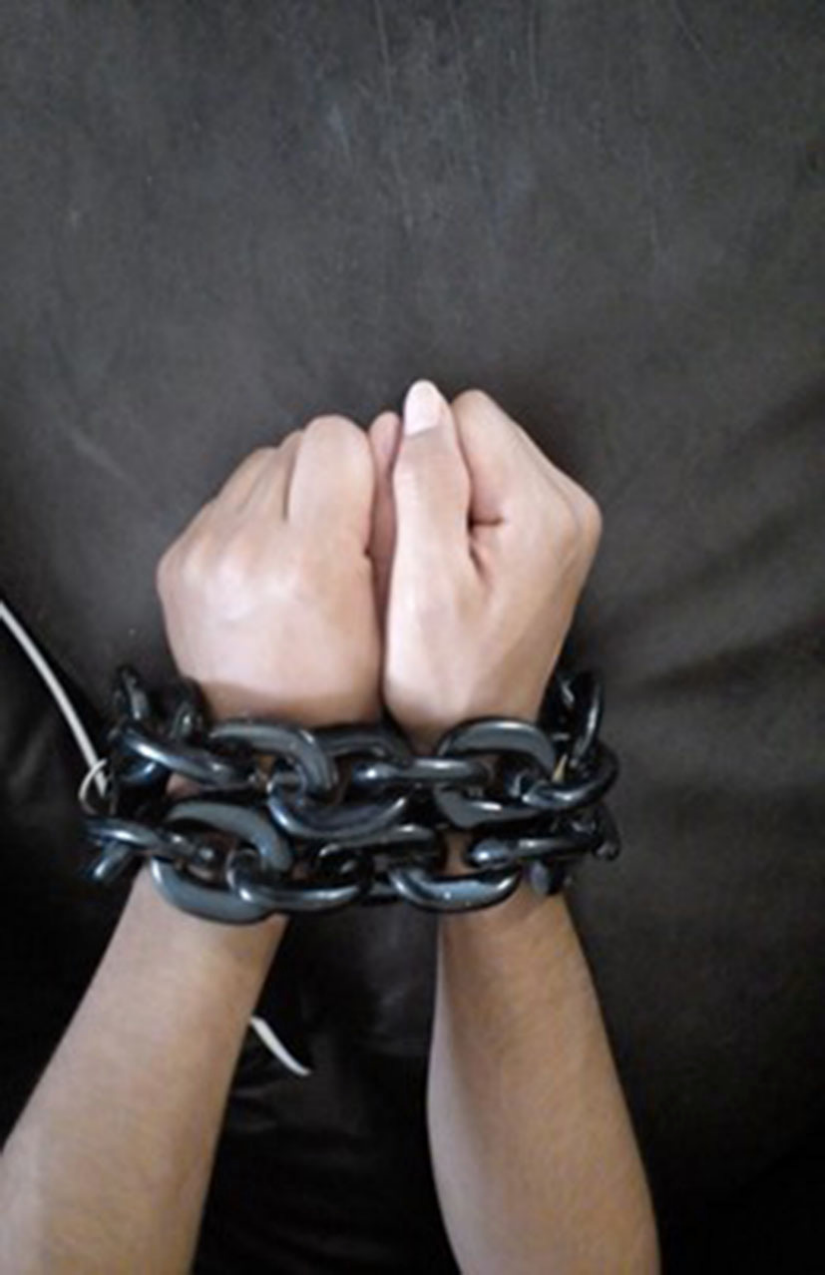


“*It represents the oppression towards certain groups of immigrants, where it is difficult to work. In our country we go through similar things; over there, if you are a trans person, you don't qualify to obtain a decent job, even if you're good at something due to your physique, because you look “feminine”. Even if you are a well-qualified person for the job, they place obstacles. In the United States, it is extremely difficult to be a foreign person, even more so if you are from Central America or stand out because of the color of your skin or because you are an immigrant. Something I would like to change would be the importance people place on how a person looks or on their color (of the skin), or in my manner of expressing myself physically, but instead place it on intellectual capacity. Give the opportunity to at least try out a person at a job”* (Figure 11).

Mane, from Honduras

#### Without Options

**Figure 12:**
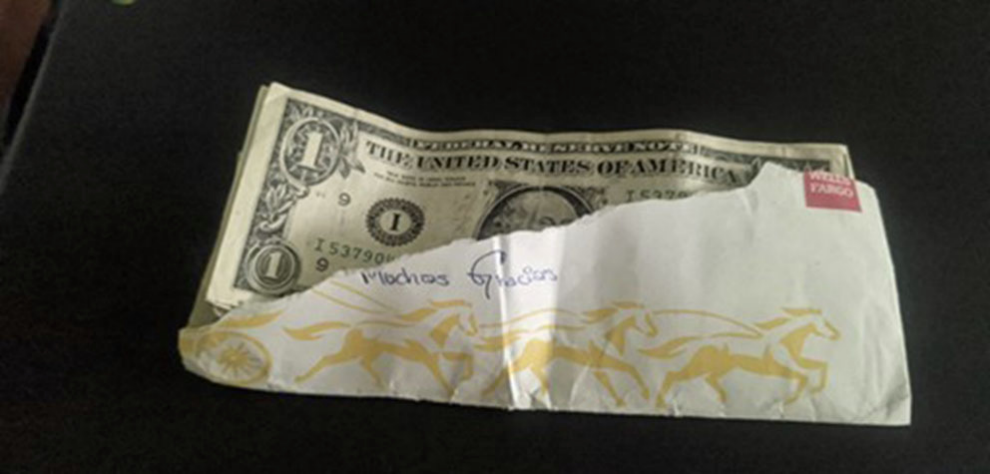


“*It is the envelope I get paid with, but what is it that is really happening? I had to make some purchases, a flight, and other things online and I really do not have other options, I do not have access to a bank, to a credit card, or to another form of access to my electronic money. I have to travel back to El Paso and I am going to have to take the money with me in cash. It makes me sad that I am unable to open an account. I do not exist yet in this country. I am a person who is worth absolutely nothing in this country. I do not like having the money in cash, it is dangerous, I can lose it, I do not know what can happen, and I do not have other options. I would like there to be a plan or a form of getting integrated into society more quickly. A work permit that could be more formal. Not to just be a person that has entered and then that is it. Because even if I wanted to produce or do something, I can't, I don't have a right to, I don't exist here yet”* (Figure 12).

Susana, from El Salvador

#### Health

**Figure 13:**
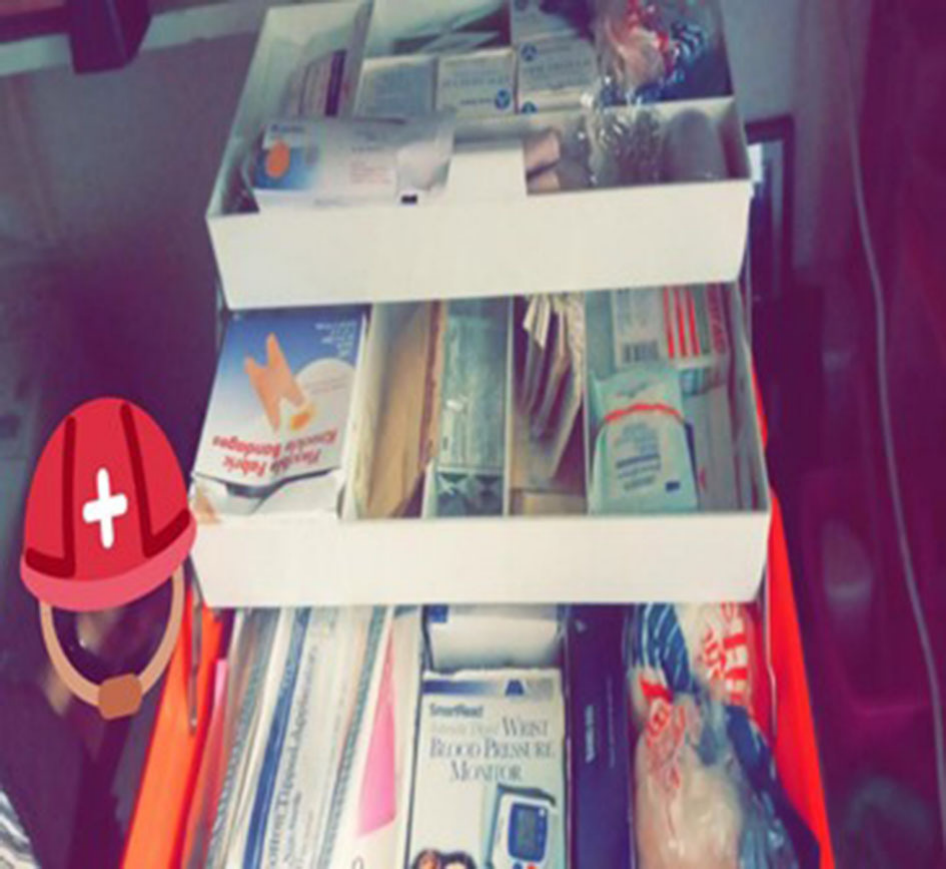


“*The health here (United States) is too expensive. If I could change something in this country, it would be to provide us with access to healthcare services. Because we arrived here with no money. How will we be able to pay such large amounts? It would be good to have some sort of basic health plan for people like us. Right now, we are okay, but if something happens to us, we are not registered or anything, if they tend to us, they are going to ask us for money and we don't have it, we are just arriving, we don't have anybody. I ask for access to health”* (Figure 13).

Ariel, from Mexico

#### More (Picture Art)

“*Bring consciousness to the work: not just because you have power does it make it okay to make others feel less than*.

*Greater knowledge of trans identity. Being trans is not being something different, it's being the same person*.

**Figure 14:**
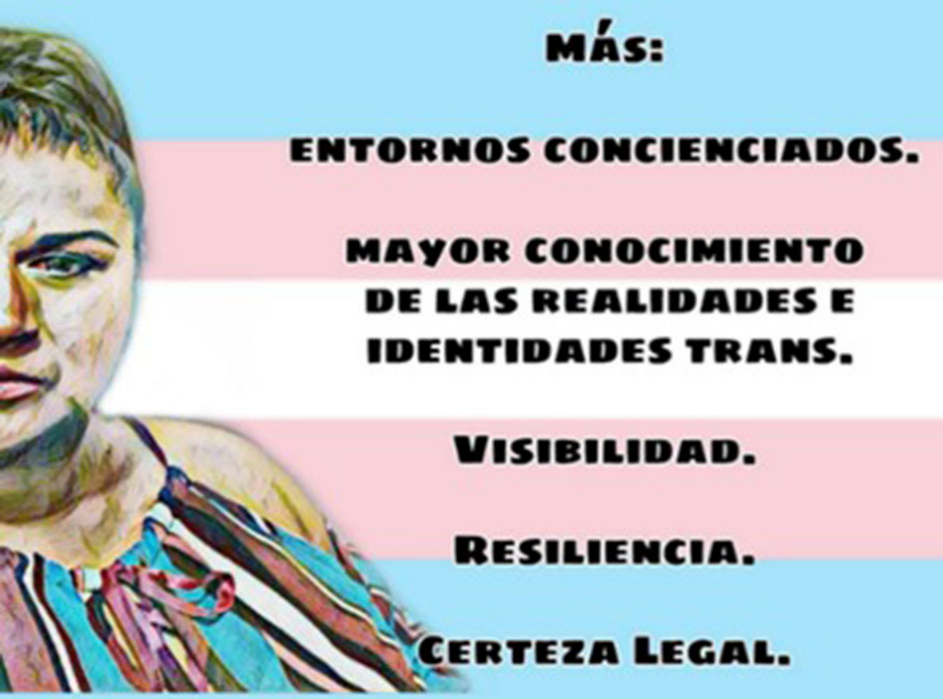


*Information is needed. Having work; for people not to discriminate; they must take into account the differences in people. Visibility for people to know that we are here, that we exist, since forever. Resilience, we need to transcend, look towards the past, and know where you come from and what you are going to do in the future. Legal certainty, “To not be from here, nor there”, to know where we are going once were are here (United States). To give continuity to the process (migratory) and inform on what can be done. We need information and gender equality. I exist!”* (Figure 14).

Lilith, from Mexico

At the end of the fourth session, after the presentation and review of the worke produced by participants, they also developed a Call to Action. This Call to Action seeks to inform policy and decision makers about the health, education and employment needs of transgender migrants. Beyond that, there are also specific recommendations to advance policy, programs and services.

### Call to Action

1. Medical insurance in a temporary form during the migratory process to be able to obtain preventive health services, medical care, medications, and mental health services.
2. Accessible and affordable education free of discrimination.
3. Dignified employment and work authorization upon entering the United States.
4. Be given the opportunity to demonstrate that we are not a public burden. On the contrary, we are valuable people who build and contribute.
5. Stop stigma, stereotypes, use of labels and all forms of work discrimination.
6. Decriminalize migrants.
7. Promote education in gender diversity early on and throughout life.
8. Legal recognition of transgender identities.
9. Ensure the right to practice a profession, occupation or job of one is choosing.
10. Guarantee the right to decide freely, autonomously and knowingly about my body, identity, and sexuality.
11. Secure a life free of violence.
12. Access to cultural and linguistic orientation and a platform of trans borderland services in the migratory process.

In addition, a bilingual documentary titled “Dignified Life” was produced to raise awareness (https://www.youtube.com/watch?v=IudYXg5W_6A&t=0s).

## Discussion

To our knowledge, this is the first study to use the Photovoice methodology on mental health transgender migrants and COVID-19, to identify interventions and program opportunities among TWC in the U.S.-Mexico Border. TWC in the study experienced mental health issues including loneliness, sadness and loss associated with forced migration. Participants also shared their desires for migration, including more freedoms in the U.S., safety, and opportunities. The majority of the participants recounted their traumatic experiences in their home country with not being able to express their gender identity with families, workplace or socially. Other participants documented the stigma, discrimination and violence toward transgender populations in their country of origin, in many instances leading to death. Other studies with TWC have documented similar challenges (38, 39). Comprehensive mental health services are crucial to support TWC. Training programs are needed to ensure bilingual mental health professionals and service providers. It is important that mental health professionals implement culturally responsive and linguistically appropriate screenings for mental health and other psychosocial conditions. Further, findings also suggest the dire need to develop multicomponent interventions. Developing, testing and scaling up interventions to support TWC in the U.S.-Mexico border could help address some of the growing health concerns affecting this population. These efforts ought to include active engagement of TWC in the development and implementation of programs and interventions.

Despite the challenges encountered in their country of origin and as immigrants in the U.S., participants highlighted resilience and resilience adaptive behaviors to cope with challenges and discriminatory experiences. Resilience has been defined as qualities or characteristics that enables/empowers individuals and communities to develop protective behaviors in the face of adversity (40). Resilience adaptive behaviors are those adaptive responses and behaviors resulting from adversity and challenges (41, 42). Researchers have described people who are developmentally thriving and people who adapt to negative circumstances as both being resilient (41, 43). Our findings of resiliency trajectories and resiliency adaptive behaviors among this population highlight the need for community-based treatment models, resilience-centered interventions, and a re-contextualization of risk in the context of TWC and historical trauma.

Given the mobility of this population, use of technologies and images, and their migration trajectories, new technologies and mobile health options have the potential to support engagement in prevention and care services. In particular, ecological momentary interventions (EMI) and assessments can facilitate changes in health behaviors and provide treatment options to patients in real time and in their natural environments (44, 45). The key feature of all EMI is that the treatment is provided to people during their everyday lives (i.e., in real time) and settings (i.e., real world). Therefore, these interventions are ecologically valid in that they occur in the natural environment at relevant moments. Using EMI content driven by Photovoice methodologies could help improve health outcomes among this population.

The majority recounted being a target by gangs. In fact, some participants recounted stories of how gang members were instructed to murder members of the transgender community prior to initiation. Because of the pervasive impact of health-harming legal needs affecting this population including being victim of violence and discrimination, we propose the uptake of medical legal partnerships (MLPs) to serve the needs of TWC. A health-harming legal need is a conflict between an individual and a government or private entity or a deprivation of a civil or legal right that directly or indirectly adversely affects a person's access to, or retention in, health care (46). Other studies have reported dramatic protective effects resulting from immigration relief among this population (38). MLPs integrate the unique civil rights law expertise into the health care setting to help clinicians, case managers, and social workers address structural problems at the root of health inequities. MLPs provide a holistic and interdisciplinary approach to patient care. There are three underlying principles in MLPs: first, the social, economic, and political contexts in which people live fundamentally impact health. Second, social and structural determinants often manifest in the form of legal needs. Social determinants shape the distribution of money, power, and resources, and legal support is often required to address resulting inequities that produce health-harming legal needs, including access to health insurance, public benefits, housing, and income support. Lastly, legal partners have the training and skills to address these legal needs. With in-depth knowledge, legal experts are uniquely situated to address health-harming legal needs impacting TWC and navigate the resultant barriers to health. Reducing or eliminating social stressors by addressing patients' legal issues can also improve health through, what Smith et al. (47) calls, the “worry budget” (47). By providing legal support to reduce social stressors, MLPs can enable TWC to prioritize health issues. Through a combination of legal support services delivered through legal partners who are positioned to operate ethically with clinics and patients, MLPs can reduce health disparities among TWC.

A binational advisory committee of decision makers and scholars reviewed a set of recommendations prepared by the project team to respond to the needs of TWC in the U.S.-Mexico Border.

### Recommendations for Implementing Public Policies and Evaluation

- Access to care as a human right to anyone despite migration status.
- Offer programs and treatments based on evidence at the place of origin to reduce migration due to violence or disparities.
- Support and develop programs to address health-harming legal needs.
- Leverage new technologies and online networks to facilitate behavioral change and engagement in prevention and care.
- Offer evidence-based programs and treatments at the host site to mitigate the impact of migration on physical and mental health, and decrease chronic degenerative and infectious diseases.
- Ensure the participation of civil society, public and foreign powers, and international institutions to formulate social protection policies.
- Measure the magnitude of transgender immigration and other needs to generate informed interventions.
- Improve living conditions by investing in educational programs for migrants.
- Develop strategies to reduce heterosexist and cissexist service delivery for all migrants as well as specialized services and programs for TWC.
- Work permits for immigrants as it provides them with economic security and allows them to feel a sense of contribution.

### Study Limitations

Some study limitations are worth mentioning. The participants consisted of a convenience sample of migrant TWC. The findings cannot be generalized to all TWC and are not representative to all migrant TWC. Despite this limitation, the findings from this study have several important implications for research, practice, and policy. The lack of mental and health services within this population underscores the need to conduct more research about the risk factors, consequences of migration, and health needs among TWC. Further, this study solely relied on the use of Photovoice methodology to generate empirical findings. Future research should consider integration of other methods of data collection including mixed-methods and longitudinal designs to further enrich findings and recommendations.

## Conclusion

Substantial health disparities and inequities continue to impact transgender women. TWC in this study highlighted unique challenges including migration experience, heterosexist obstacles and power structures, as well as personal strengths and resilience. The findings position TWC as determined, hopeful and resilient. Through their stories, they highlighted their experiences challenging normative discourses and dignified life of opportunities free of violence. Recommendations include the need to advance human sexuality education and rights as well as public policies and structural interventions in a way that offers all people the rights and recognition that they deserve. As part of the conclusions and lessons learned after the study realization, the authors identified a series of recommendations for public policies to advance human rights and mitigate inequities.

## Data Availability Statement

Data supporting the findings of this study are available from the corresponding author on request.

## Ethics Statement

This study was carried out in accordance with the recommendations of the University of Texas at El Paso, Institutional Review Board committee' with written informed consent from all subjects. All subjects gave written informed consent in accordance with the Declaration of Helsinki. The protocol was approved by the University of Texas at El Paso IRB committee. The patients/participants provided their written informed consent to participate in this study. Written informed consent was obtained from the individual(s) for the publication of any potentially identifiable images or data included in this article.

## Author Contributions

EM and SC-B co-led the design of research, acquisition of information, drafts and revisions of the paper, and agreed to be accountable for all aspects of the work. OM member of the research team and project advisory committee member, provided advice on study design and analysis, and agreed to be accountable for all aspects of the work. PC participated in the conceptualization of the study protocol, research design, and procedures and analysis. All authors contributed to the article and approved the submitted version.

## Funding

Research reported in this publication was supported by the National Institute on Minority Health and Health Disparities of the National Institutes of Health under Award Number U54MD007592. The content is solely the responsibility of the authors and does not necessarily represent the official views of the National Institutes of Health.

## Conflict of Interest

The authors declare that the research was conducted in the absence of any commercial or financial relationships that could be construed as a potential conflict of interest.

## Publisher's Note

All claims expressed in this article are solely those of the authors and do not necessarily represent those of their affiliated organizations, or those of the publisher, the editors and the reviewers. Any product that may be evaluated in this article, or claim that may be made by its manufacturer, is not guaranteed or endorsed by the publisher.
